# Turkish graveyards as refuges for orchids against tuber harvest

**DOI:** 10.1002/ece3.3562

**Published:** 2017-11-23

**Authors:** Attila Molnár V., Timea Nagy, Viktor Löki, Kristóf Süveges, Attila Takács, Judit Bódis, Jácint Tökölyi

**Affiliations:** ^1^ Department of Botany University of Debrecen Debrecen Hungary; ^2^ Department of Plant Sciences and Biotechnology Georgikon Faculty University of Pannonia Keszthely Hungary; ^3^ MTA‐DE “Lendület” Evolutionary Phylogenomic Research Group University of Debrecen Debrecen Hungary; ^4^ MTA‐DE Behavioural Ecology Research Group University of Debrecen Debrecen Hungary

**Keywords:** Asia minor, cemetery, CITES, Orchidaceae, salep, sustainability

## Abstract

Harvest of orchid tubers for salep production is widespread in southwestern Asia and the Balkans and constitutes a major conservation risk for wild orchid populations. Synanthropic habitats, such as graveyards, are important refuges for orchids and other organisms and could offer protection from salep harvesting because of their special cultural role. However, little is known about the occurrence and factors influencing harvesting of salep in graveyards. During field surveys of 474 graveyards throughout Turkey, we observed 333 graveyards with orchids, 311 graveyards with tuberous orchids, and salep harvest in 14 graveyards. Altogether, 530 individuals of 17 orchid species were collected, representing 9% of the individuals recorded. Harvesting intensity was relatively low, and populations were usually not wholly destroyed. However, some species were clearly more affected than others. Salep harvesting risk of orchid species was significantly associated with flowering time, with early‐flowering species being more affected. A marginally significant positive relationship between harvesting risk and species‐specific tuber size was also detected. Our data suggest that graveyards might offer some protection against salep harvesting in Turkey, but they also show that some orchid taxa are much more affected than others. Overall, our observations add more weight to the conservation value of these special habitats.

## 1 INTRODUCTION

Harvesting tubers of terrestrial orchids to obtain a hot winter beverage (“*salep”*) or a special type of ice cream (“*salepi dondurma*”) is a century‐old, widespread practice in Turkey (Sezik, 2002a,b; Tamer, Karaman, & Copur, 2006) and the Balkans (Kreziou, de Boer, & Gravendeel, 2015; Matović, Nikolić, Đelić, & Marković, 2010) and is recently booming in Iran as a result of increased demands from Turkey (Ghorbani, Gravendeel, Naghibi, & de Boer, 2014; Ghorbani, Gravendeel, Selliah, Zarré, & de Boer, 2017).

**Figure 1:**
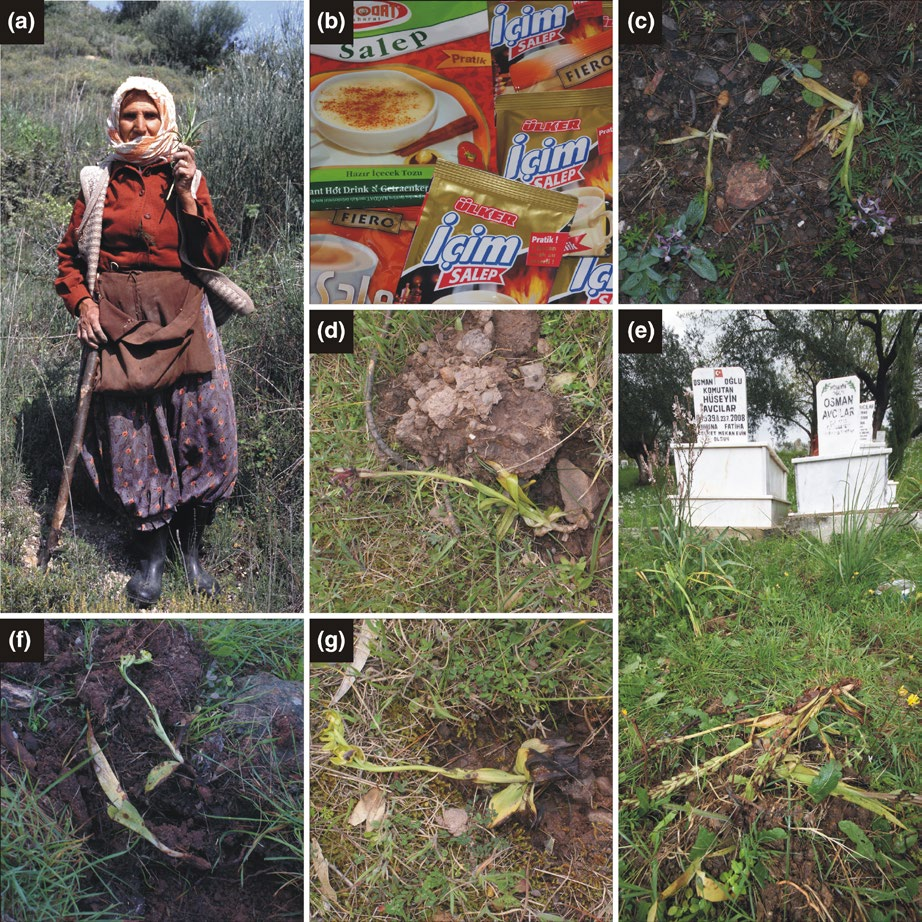
(a) Salep harvesting elderly woman near Söke (Aydın) in April 1993; (b) different instant salep products are widely available in Turkish stores; (c) excavated *Anacamptis syriaca* specimens in the graveyard of Belen village (Antalya Province); (d) excavated flowering *Anacamptis papilionacea* specimen in the graveyard of Bayır village (Muğla Province); (e) excavated fruiting *Himantoglossum robertianum* specimens in the graveyard of Kemer village (Muğla Province); (f) excavated juvenile *H. robertianum* individual and flowering *Ophrys lutea* subsp. *minor* specimen in the graveyard of Beşikci village (Antalya Province); (g) excavated *Ophrys blithopertha* specimen in the graveyard of Bayır (Muğla Province)—photographs: a–d, f–g by A. Molnár V.; E by V. Löki

Salep harvesting—along with habitat loss, intensification of agricultural land use (Şekercioğlu et al., 2011; Yilmaz, 1997), and overgrazing (Özhatay, Koçyiğit, Yüzbaşıoğlu, & Gürdal, 2013)—is considered as a major factor threatening Turkey's diverse and unique orchid flora (Kasparek & Grimm, 1999; Kreutz, 1998; Sezik, 2002b, 2006; Tecimen et al., 2010). During salep harvesting, new (daughter) tubers of orchids are removed mostly in their generative state (Tamer et al., 2006), thereby destroying the affected individuals (Figure 1a,c–e). Sezik (2002a) considers that 85% of orchid species are affected by salep harvesting, while Tamer et al. (2006) report that there are 90 orchid species belonging to 24 genera used in salep production in Turkey.

The estimation of inland trade is nearly impossible, but the exported amount increased continuously since the 1990's; in 1993, it reached 75,100 kg in a year, and according to official Turkish statistics, at least 28,200 kg of salep was exported annually between 1994 and 1999 (Kasparek & Grimm, 1999). To gain 1 kg of dried salep, approximately 625–4,762 specimens (mean ± *SD* = 2,599 ± 1,710) are destructively harvested (Sezik, 2002b). The number of orchid individuals collected annually in Turkey is estimated at 10–20 million by Kasparek and Grimm (1999), 30 million by Özhatay (2002), and 40 million by Sezik (2002a) Sezik (2002b).

**Figure 2:**
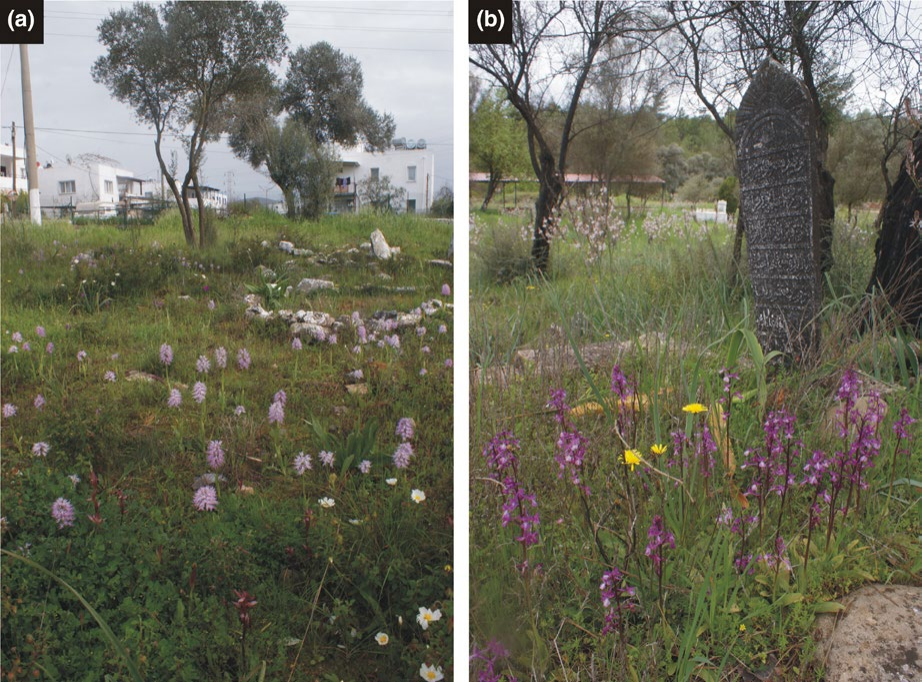
Mass occurrences of orchids in Turkish graveyards. (a) graveyard of Kızılağaç village (Muğla) with *Orchis italica*, (b) graveyard of Uğrar village (Antalya) with *Orchis anatolica*. Photographed by A. Molnár V

The increased wealth of the middle class, and the growing western export resulted in increased demand for salep, and its price also increased substantially (Ghorbani et al., 2014, 2017). As a consequence, the unsustainable collection of tubers threatens wild orchid populations (cultivating terrestrial orchids for salep production is not known). To develop a useful method and routine for salep collecting, it would be essential to know more about patterns of collection (Erzurumlu & Doran, 2011), the species most affected, levels of sustainable harvesting (Sandal & Söğüt, 2010), and types of intervention which could effectively control the salep trade (Entwistle, Atay, Byfield, & Oldfield, 2002). Furthermore, preserving remaining orchid populations is essential until suitable harvesting practices are developed using education of local people (Light, Kell, & Jackson, 2003), development of effective legislation (Kasparek & Grimm, 1999), designation of protected areas (Ghorbani et al., 2014), or applying indigenous bulb propagation of orchid species traditionally used for salep to substitute collecting orchids from nature (Tekinşen & Güner, 2010). Burial places are increasingly recognized as valuable habitats for biodiversity conservation worldwide. In a single urban cemetery from Berlin, for instance, Kowarik, Buchholz, von der Lippe, and Seitz (2016) detected 604 animal and plant species including bats, birds, lichens, bryophytes, carabids, vascular plants, and spiders. An increasing number of studies report high plant and animal species richness in graveyards throughout the world (e.g., Aerts et al., 2016; Ahmed et al., 2009; Čanády & Mošanský, 2017; De Lacy & Shackleton, 2017; Gao, Ouyang, Chen, & van Koppen, 2013; Latta, Musher, Latta, & Katzner, 2013; Löki et al., 2015), and many of these graveyards, cemeteries, or sacred forests contain vulnerable, threatened, or endangered species that occur less frequently in other urban ecosystems or were thought to be extinct in the surrounding area (Kowarik et al., 2016; Molnár V., Löki et al., 2017; Özhatay & Gürdal, 2013). Graveyards can preserve species or entire communities from the original habitats when the surrounding landscape becomes degraded, such as in the case of threatened vascular plants of North American prairies (Could, 1941), Australian grassy white box woodlands (Prober, 1996), steppe plants in the Pannonian region (Molnár V., Löki et al., 2017), or medicinal plants in Pakistan (Hadi, Ibrar, & Zaidi, 2014). Furthermore, these special habitats can act as corridors of dispersal for some organisms (Munshi‐South, 2012). In the midst of the changing socioeconomical and natural conditions in Turkey, it is recognized that graveyards can play a significant role in conserving orchids of Turkey (Figure 2). Botanist and amateur orchid enthusiasts recognized decades ago that orchids regularly occur in Turkish graveyards (Kaya, Varol, & Aytepe, 2008; Kreutz, 1998; Kreutz & Çolak, 2009; Sundermann & Taubenheim, 1978). A comprehensive field survey of orchids in Turkish graveyards was carried out recently; this study also demonstrated that salep harvesting does occur in Turkish graveyards (Löki et al., 2015). However, the actual amount of the collected species, the number of collected individuals, and generally the collecting preferences of local people are unknown.

Our aims in this study were (1) to comprehensively document salep harvesting activity in Turkish graveyards and (2) identify factors that might affect salep harvesting risk in orchid taxa. We hypothesized that specific traits of tuberous orchids, such as conspicuousness, tuber size, and flowering phenology, might predict salep harvesting. Conspicuousness of different orchids can be very different as a consequence of variation in height of flowering shoot. Mean tuber size is also highly variable between species. Specific variability of tuber size causes substantial differences in average weight of dried tubers of salep originated in different regions of Turkey (Sezik, 2002b), characterized by different orchid species composition. As the size of tubers can potentially be important for the salep harvesters, it could affect harvesting preferences. Salep harvest is limited to a relatively short (ca. 1‐month‐long) period (Molnár V., Süveges, Molnár, & Löki, 2017; Sezik, 2002a); therefore, we hypothesized that specific flowering phenological characteristics are also important in shaping salep harvesting preferences.

## 2 MATERIALS AND METHODS

### 2.1 Fieldwork and parameters of graveyards

**Figure 3:**
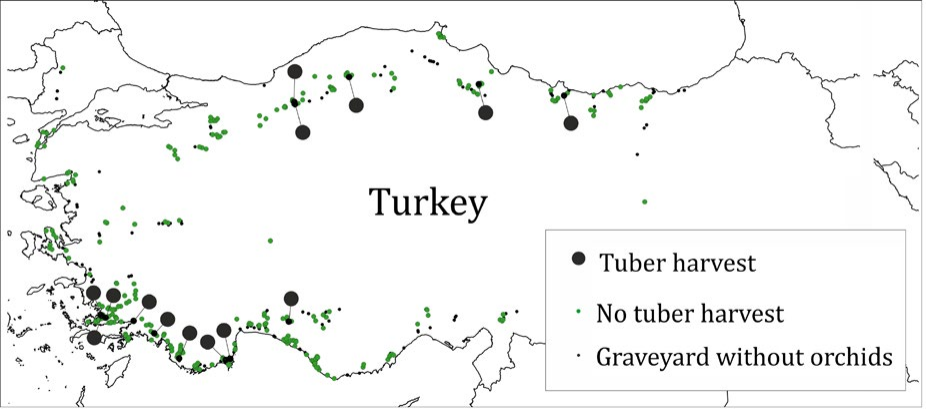
Salep harvesting activities recorded and graveyards studied

We surveyed 455 Muslim burial grounds (Turkish: mezarlık, hereafter graveyards) regardless of their spatial dimension, position within settlements, or presence of religious facilities in 2 years: 300 graveyards have been evaluated in 2014 (Löki et al., 2015) and 174 in 2015 (Table S1; Figure 3). We visited 19 graveyards in both years (one in Balıkesir, 13 in Muğla, and five in Antalya provinces). We recorded the altitude and geocoordinates of all visited graveyards (Table S1) by Garmin eTrex Legend handheld device. The visited graveyards were systematically searched for orchids, including excavated individuals (Figure 1c–e). Because salep harvesters generally collect only newly developed (daughter) tubers and leave the remaining plant parts, we were able to confidently identify affected individuals at specific level in most cases. We followed the nomenclature used in Kreutz and Çolak (2009), except in the case of the genus *Himantoglossum* Spreng. s.l. (incl. *Barlia* Parl. and *Comperia* K. Koch), where we followed the nomenclature of Sramkó, Molnár V., Hawkins, and Bateman (2014).

### 2.2 Quantification of species traits

We quantified the length and width of the new tuber and the height of flowering stem from herbarium specimens (Table S2) using ImageJ 1.4.3.67 software. We used 864 digitized herbarium individuals of 51 species in 17 natural history collections [BAS (UK), BASBG (Switzerland), BOD (UK), BP (Hungary), DE (Hungary), EGE (Turkey), GAZI (Turkey), HUB (Turkey), HUEF (Turkey), ISTE (Turkey), IZEF‐NR (Turkey), MUH (Pakistan), NGBB (Turkey), RBGE (UK), RENZ (Switzerland), W (Austria), and WU (Austria)]. From length and width of the new tuber, we calculated ellipsoid volume to obtain three‐dimensional estimates from herbarium tubers. In case of seven *Ophrys*, one *Himantoglossum,* and one *Serapias* species, we did not find enough measurable herbarium specimens, and we assigned the average of the measured traits at generic level to these species.

Average flowering time of orchids was obtained from flowering intervals published in Kreutz and Çolak (2009). These data are given with a precision of approximately 10 days (thirds of a month). We assigned a sequential number from 1 (first third of January) to 36 (last third of December) to these periods. Species‐specific flowering time was calculated as the average of the beginning and end of the flowering interval. For example, the flowering period of *Anacamptis pyramidalis* (L.) L. C. M. Rich. lasts from beginning of April (10) to mid‐July (20); hence, average flowering time of this species is 15.

Excavated specimens of *Anacamptis* and *Ophrys* in vegetative stage which were unidentifiable at the species level we excluded from the analyses.

### 2.3 Data analyses

To understand which species characteristics affect salep harvesting, we used data from 14 graveyards in which salep harvesting was observed. For these graveyards, the number of harvested and unharvested orchids (treated separately for each species) was used as a bivariate response in a binomial Generalized Linear Mixed Model (GLMM). Plant height, volume of new tubers, and the average flowering time were included as explanatory variables. We also included genus as a random factor in these models to take into account the fact that closely related species are more similar to each other than expected by chance (i.e., there is phylogenetic inertia). Explanatory variables were Box‐Cox transformed and standardized to optimize model fit. We removed nonsignificant predictors from the complete model in a stepwise manner (based on the largest p‐values) in order to get a minimal model which contained only significant predictors. All models were built in the R Statistical Environment (R Core Team 2017).

## 3 RESULTS

We found orchids in 208 of 300 visited graveyards in 2014 (electronic supplement of Löki et al., 2015) and 124 of 174 graveyards in 2015 (Table S1). We found tuberous orchids (potentially affected by salep harvesting) in 311 of 455 graveyards (68.3%). In two cases, taxonomic identity could only be assigned at the genus level, because only basal leaf rosettes were observable (*Anacamptis* sp., *Ophrys* sp.). We observed salep harvesting activity only in 14 graveyards (4.5%) of 311 graveyards hosting tuberous orchids (Table 1, Figure 3). The collection of tubers affected 530 individuals of 17 species, (Table 2), belonging to three genera (*Anacamptis*: 44.4% of collected individuals, *Himantoglossum*: 39.5%, and *Ophrys*: 16.0%). The highest frequency of salep collection in graveyards (in eight cases) was observed in Muğla province. The number of excavated individuals in a graveyard varied from 6 to 172. *Himantoglossum robertianum* (Lois.) P. Delforge (159 individuals) and *Anacamptis pyramidalis* (152) were collected in the highest individual number, while *Himantoglossum robertianum* (six graveyards), *Himantoglossum jankae* Somlyay, Kreutz, and Óvári (3), *Anacamptis pyramidalis* (3), and *Ophrys holoserica* subsp. *heterochila* Renz and Taubenheim (3) were harvested in most graveyards. The number of harvested species in a graveyard varied from 1 to 5. In those graveyards where salep harvesting occurred, mean ± *SD* = 37.0 ± 20.8% of species were excavated. In two graveyards, each visible individual of *H. robertianum* was excavated. This species was collected in both studied years in the settlements of Kemer and Meşelik (Muğla); in 2014, 50% of the individuals were removed, while in 2015, 100% in Meşelik, and 94% in Kemer was removed (Table 1). We are reporting *Ophrys subfusca* subsp. *blithopertha* (Paulus & Gack) Kreutz as a new taxon for salep harvesting from the graveyard of Bayır (Muğla, 2015, Figure 1g). Additionally, a previously unknown collecting habit was observed during our field survey: both tubers had been removed from vegetative individuals of *Anacamptis pyramidalis* in the graveyard of Akyaka (Muğla).

**Table 1:** Graveyards with salep harvesting. The total number of recorded individuals and the number of harvested specimens are given in parentheses

No.	Locality	Province	Location	Alt. (m)	Year	Harvested taxa
213	Meşelik	Muğla	37.15852°N, 27.58838°E	100	2014	*Himantoglossum robertianum* (12/6)
213	Meşelik	Muğla	37.15852°N, 27.58838°E	100	2015	*Himantoglossum robertianum* (8/8)
209	Kemer	Muğla	37.13983°N, 27.61466°E	27	2014	*Himantoglossum robertianum* (20/9), *Ophrys speculum* var. *orientalis* (10/3)
209	Kemer	Muğla	37.13983°N, 27.61466°E	27	2015	*Himantoglossum robertianum* (53/50), *Ophrys umbilicata* (3/1), *Anacamptis sancta* (50/10), *Ophrys tenthredinifera* subsp. *villosa* (8/8)
199	Çukurincir	Muğla	36.39403°N, 29.31937°E	32	2014	*Anacamptis coriophora* subsp. *fragrans* (16/16)
10	Belen	Antalya	36.38612°N, 30.44489°E	50	2014	*Ophrys candica* var. *minoa* (200/2), *Anacamptis* subsp. *syriaca* (45/10)
16	Emiraşıklar	Antalya	37.04133°N, 31.73143°E	935	2014	*Anacamptis pyramidalis* (200/2)
59	Afşar	Bolu	40.74631°N, 31.86908°E	980	2014	*Himantoglossum jankae* (52/10)
222	Cevizlik	Ordu	40.88968°N, 37.78910°E	421	2014	*Anacamptis pyramidalis* (400/50)
250	Alaçamderesi	Samsun	41.07878°N, 35.91288°E	790	2014	*Himantoglossum caprinum* (3/2), *Himantoglossum comperianum* (6/1), *Himantglossum jankae* (6/1)
140	Damla	Kastamonu	41.19473°N, 33.05998°E	964	2014	*Himantoglossum jankae* (6/5)
77	Yayladınlar	Bolu	40.78555°N, 31.85373°E	775	2014	*Himantoglossum jankae* (65/19)
195	Akyaka	Muğla	37.05373°N, 28.31655°E	29	2015	*Anacamptis pyramidalis* (400/100), *Ophrys amanensis* subsp. *antalyensis* (14/2)
454	Tepearası	Muğla	36.83469°N, 28.77213°E	17	2015	*Anacamptis* sp. (100/13), *Ophrys holoserica* subsp. *heterochila* (20/2), *Ophrys* sp. (2/1)
14	Beşikci	Antalya	36.36651°N, 30.34113°E	92	2015	*Himantoglossum robertianum* (40/16), *Himantoglossum comperianum* (20/2), *Anacamptis morio* subsp. *syriaca* (500/5), *Ophrys holoserica* subsp. *heterochila* (30/1), *Ophrys lutea* subsp. *minor* (20/3)
400	Bayır	Muğla	37.10906°N, 27.70012°E	161	2015	*Himantoglossum robertianum* (70/70), *Ophrys subfusca* subsp. *blitopertha* (1/1), *Anacamptis papilionacea* subsp. *messenica* (160/40), *Ophrys holoserica* subsp. *heterochila* (120/60), *Ophrys tenthredinifera* subsp. *villosa* (10/1)

**Table 2:** Summary of recorded salep harvesting activities

No.	Locality	Year	Number of collected taxa	Total number of collected individuals	Number of observed orchid taxa	Total number of observed individuals	Proportion of collected taxa (%)	Proportion of collected specimens (%)
213	Meşelik	2014	1	6	6	81	17	7
213	Meşelik	2015	1	8	5	27	20	30
209	Kemer	2014	2	12	8	133	25	9
209	Kemer	2015	4	69	6	145	67	48
199	Çukurincir	2014	1	16	6	246	17	7
10	Belen	2014	2	12	9	806	22	1
16	Emiraşıklar	2014	1	2	10	907	10	1
59	Afşar	2014	1	10	5	243	20	4
222	Cevizlik	2014	1	50	3	429	33	12
250	Alaçamderesi	2014	3	4	7	115	43	3
140	Damla	2014	1	5	4	59	25	8
77	Yayladınlar	2014	1	19	2	299	50	6
195	Akyaka	2015	2	102	3	454	67	22
454	Tepearası	2015	3	16	4	127	75	13
14	Beşikci	2015	5	27	9	1,265	56	2
400	Bayır	2015	5	172	11	563	45	31

**Figure 4:**
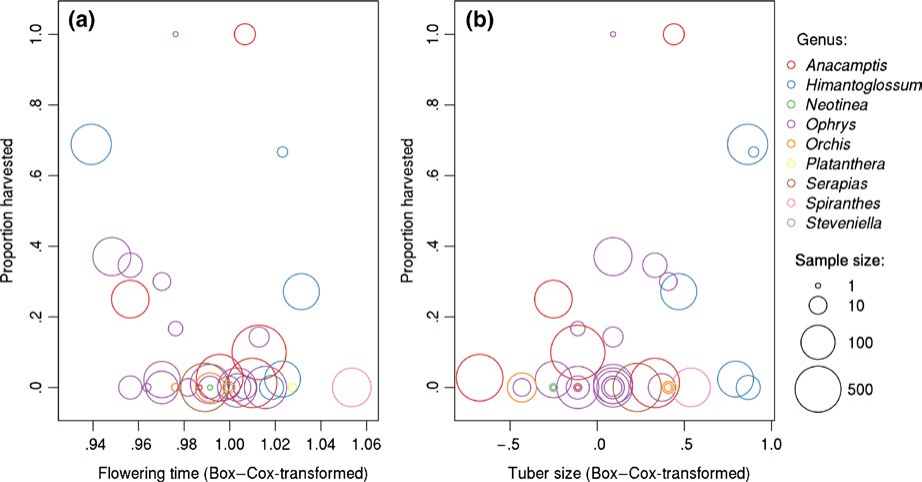
Connection of flowering time (a) and tuber size (b) with species‐specific harvesting probability detected in Turkish graveyards

Salep harvesting risk was significantly negatively related to average flowering time (Table 3, Figure 4b), implying that early‐flowering taxa were harvested more frequently. We also found a marginally significant positive relationship between harvesting frequency and tuber size (Table 3, Figure 4b). However, this variable dropped out during the model simplification procedure.

**Table 3:** Full and minimal GLMMs built to explain species‐specific harvesting frequency of orchids in Turkish graveyards. All explanatory variables were Box‐Cox transformed and standardized to improve model fit

	Full model		Minimal model	
	Estimate (*SE*)	*p*‐Value	Estimate (*SE*)	*p*‐Value
Height of flowering stem	**−**0.456 (0.977)	.641		
Flowering time	**−**1.214 (0.617)	.050	**−**1.282 (0.601)	.033
Tuber size	1.575 (0.903)	.082		

## 4 DISCUSSION

Salep harvesting is a major threat for orchids in Turkey, but few studies have systematically explored patterns of tuber collection and variation in collection risk among species. We hypothesized that graveyards might offer protection from salep harvesting because of their special sociocultural role, which might prevent digging activity. Contrary to this expectation, our results show that graveyards are not free of salep harvesting. However, several lines of reasoning (elaborated below) suggest that harvesting intensity might be relatively low in graveyards, thereby providing some degree of protection to orchids inhabiting these seminatural anthropogenic habitats.

First, although salep harvesting is widespread in Turkey, we detected excavation of orchid tubers in only 14 graveyards (4.5%) of a sample of 311 graveyards hosting tuberous orchids. As graveyards are places that are relatively highly frequented by the local inhabitants, this low intensity likely indicates lower preference for harvesting at these sites, rather than reduced detection ability. Second, in graveyards where salep collection was observed, the proportion of harvested individuals was nearly always smaller than the full population size, indicating that not all individuals were harvested (this could occur, e.g., if collectors avoid digging in the vicinity of graves). Third, compared to the average salep harvesting activity required for an economically sufficient amount of profit (Ghorbani et al., 2014; Kasparek & Grimm, 1999; Özhatay, Koyuncu, Atay, & Byfield, 1997), the amount collected in graveyards was quite small. As a consequence of the above factors, viable populations may survive in these anthropogenically influenced habitats despite some salep harvesting activity. For instance, we found strong populations of 10 orchid species (and limited salep harvesting activity) in one of the graveyards of Emiraşıklar in 2010 and 2014, where Wagner reported in 1996 that “every single orchid has been excavated for salep purposes, only the fresh holes were visible in the area” (Kreutz, 1998: 128). At the scale of harvesting detected in this study, tuber collection is probably not the most important threat to orchids in Turkish graveyards. Instead, other anthropogenic factors, such as modern management practices (e.g., removing of native woody elements of original vegetation and extensive use of herbicides), pose a much greater concern (Molnár V. et al., unpublished data.).

While collection intensity appeared to be relatively low in graveyards, we also found that orchid species are not equally affected by harvesting: relative to their occurrence in graveyards, some species were proportionally much more affected than others. All collected individuals belonged to three genera (*Anacamptis, Himantoglossum,* and *Ophrys*). The high frequency of harvested *Himantoglossum* individuals (39.5%), and the low frequency of *Orchis* (0%) within our sample of harvested individuals, contrasts with a previous study from Iran (Ghorbani et al., 2017). There are several, mutually nonexclusive explanations for this discrepancy. First, the availability of these orchid taxa might differ between study sites, potentially affecting their harvesting frequency. Second, harvesting preferences might differ between countries. Third, it is possible that harvesting activity is altered in graveyards, for example, because harvesters attempt to keep disturbance at a minimum and collect only individuals of highly prized taxa. To find out whether the species composition of harvested individuals described in this study is typical for other habitats as well, more data on salep harvesting from outside graveyards would be strongly required.

Within the sample of harvested individuals recorded in this study, the probability of being harvested was higher in early‐flowering species. The relationship between flowering time and harvesting risk makes sense based on previous knowledge about salep collection, which seems to be restricted to a relatively short period during the spring (Molnár V., Süveges et al., 2017; Sezik, 2002a), possibly because orchids are more easily detected at this time and/or tubers are in a better condition (i.e., containing sufficient nutrients for salep production). This latter explanation is supported by the fact that salep harvesters are generally collecting only the fresh, hard, recently developed new tubers, and they leave old tubers (Kasparek & Grimm, 1999; an exception was detected during our survey in which both tubers have been removed from excavated specimens before flowering). Because of this unequal harvesting, early‐flowering taxa are probably at a higher risk from salep collection than late‐flowering ones. We also found a marginally significant relationship between tuber size and harvesting frequency, which suggests that species with large tubers might be at a higher risk of being harvested. However, more data are required to clearly ascertain this relationship. Furthermore, it remains to be shown whether these relationships hold for orchids harvested outside graveyards, where harvesting activity might be different.

Based on our data, Turkish graveyards still host diverse orchid flora and represent important orchid habitats, despite the detected salep harvesting activity. Our results strengthen the emerging view that graveyards may play an important role for diversity conservation not only in large cities (e.g., McPherson & Nilon, 1987; Kocian, Némethová, Melicherová, & Matusková, 2003; Munshi‐South 2012; Latta et al., 2013; Butt, Lowe, & Duncanson, 2014; Buchholz et al., 2016; Čanády & Mošanský, 2017) but also when the surrounding land cover has been extensively transformed (McBarron, Benson, & Doherty, 1988; Ruch, Torke, Badger, & Rothrock, 2014). Our results also emphasize the special cultural–funerary role of graveyards in reducing the impacts of human exploitation on natural resources.

## 5 IMPLICATIONS FOR MANAGEMENT

Due intensification of agriculture and rapidly changing land use, the role and significance of graveyards in conservation of living natural heritage of Turkey will probably grow. Therefore, following disposes promoting long‐term maintenance of viable and valuable populations of orchids (and additionally other different organisms) are essentially required: (1) supporting awareness of sociocultural and conservational importance of graveyards among Turkish public; (2) In subsidizing of long‐term survival of orchids (as conservational flagship species) it is especially important to keep on at least on recent low level of salep harvesting in graveyards or even reducing its intensity; (3) our dataset may help in designation of most important graveyards in orchid conservation. National official protection or at least local council protection of graveyards hosting more than five orchid species can be recommended; (4) Development of field and/or tissue culture cultivation of orchids is highly recommended to satisfy of increasing (partly foreign) commercial salep demand in favor and saving wild orchid populations. Based on our results, the highest yield is expected from the cultivation of the largest tuberous orchids (especially *Himantoglossum* spp.). In a wider outlook, enhancing long‐established burial practices and traditional management of Turkish graveyards (including minimization of human intervention) may allow survival of natural vegetation.

## CONFLICT OF INTEREST

The authors declare that they have no conflict of interest.

## AUTHORS' CONTRIBUTIONS

AMV conceived and designed the study; AMV, TN, VL, KS, and AT collected the data; TN and JT performed the analyses; JT, TN, and AMV led the writing with contributions from all authors.
