# Supplementary material for: Turkish graveyards as refuges for orchids against tuber harvest
Source: Ecol Evol. 2017 Nov 23;7(24):11257–64. doi: 10.1002/ece3.3562 (PMC5743569; doi:10.1002/ece3.3562)
Supplement: Supplementary file 1 [file ECE3-7-11257-s001.doc]

**Turkish graveyards as refuges for orchids against tuber harvest**

**Supplementary material**

Electronic appendix

**Table S1**

Numbering, geographic location, altitude, area, and orchid taxa of 155 graveyards studied in 2015, which were previously unvisited in 2014. Localities are listed alphabetically, first by province, then by settlement. An asterisk “*” indicates the 34 graveyards known formerly as orchid locations. A dash “–” indicates that no orchid taxa were recorded.

| **Nr.** | **Settlement** | **Province** | **Geocoordinates** | **Altitude (m)** | **Orchids** |
| --- | --- | --- | --- | --- | --- |
| 301 | Adrasan | Antalya | 36.33249°N, 30.43427°E | 53 | *Anacamptis* sp. |
| 302 | Ağullu | Antalya | 36.21908°N, 29.69104°E | 482 | – |
| 303 | Ahatlı | Antalya | 36.26444°N, 29.73190°E | 391 | – |
| 304 | Aksu | Antalya | 36.94054°N, 30.82463°E | 110 | – |
| 305 | Antalya | Antalya | 36.90931°N, 30.66139°E | 72 | – |
| 306 | Aydıncık | Antalya | 36.31108°N, 32.31395°E | 51 | *Limodorum abortivum*, *Ophrys holoserica* subsp. *heterochila*, *Ophrys mammosa* subsp. *posteria*, *Spiranthes spiralis* |
| 307 | Aydıncık | Antalya | 36.30357°N, 32.30612°E | 51 | *Limodorum abortivum*, *Ophrys holoserica* subsp. *heterochila*, *Serapias bergonii* subsp. *politisii* |
| 308 | Belen | Antalya | 36.37956°N, 30.38781°E | 519 | *Anacamptis pyramidalis*, *Himantoglossum robertianum*, *Ophrys lutea* subsp. *minor*, *Ophrys reinholdii*, *Serapias bergonii* subsp*. politisii*, *Spiranthes spiralis*, |
| 309 | Beykonak | Antalya | 36.34001°N, 30.32938°E | 27 | – |
| 310 | Beymelek | Antalya | 36.28259°N, 30.04316°E | 41 | *Anacamptis sancta*, *Spiranthes spiralis* |
| 311 | Boğazcık | Antalya | 36.19221°N, 29.75237°E | 285 | – |
| 312 | Burçaklar | Antalya | 36.68940°N, 31.89422°E | 731 | *Anacamptis* sp., *Limodorum abortivum*, *Ophrys holoserica* subsp. *heterochila*, *Ophrys mammosa* subsp*. posteria*, *Spiranthes spiralis* |
| 313 | Burmahancı | Antalya | 36.95656°N, 31.07376°E | 15 | *Serapias bergonii* subsp*. politisii* |
| 314 | Çakallar 1/1 | Antalya | 36.68874°N, 31.72644°E | 75 | – |
| 315 | Çakallar 1/2 | Antalya | 36.69068°N, 31.74087°E | 119 | *Ophrys mammosa* subsp. *posteria* |
| 316 | Çamyuva | Antalya | 36.56128°N, 30.55496°E | 17 | *Limodorum abortivum* |
| 317 | Çandır | Antalya | 36.97348°N, 31.05199°E | 21 | – |
| 318 | Çavuşköy* | Antalya | 36.72554°N, 31.61926°E | 7 | *Ophrys lutea* subsp*. minor*, *Ophrys mammosa* subsp. *posteria*, *Serapias bergonii* subsp*. politisii*, *Spiranthes spiralis* |
| 319 | Çerler | Antalya | 36.28691°N, 29.78298°E | 437 | – |
| 320 | Çobanlar | Antalya | 36.23338°N, 32.37058°E | 57 | *Serapias bergonii* subsp. *politisii* |
| 321 | Davazlar | Antalya | 36.26088°N, 29.87809°E | 558 | – |
| 322 | Dere | Antalya | 36.26177°N, 29.66319°E | 449 | *Anacamptis* sp., *Himantoglossum robertianum*, *Orchis simia*, *Ophrys* sp. |
| 323 | Dere | Antalya | 36.26923°N, 29.66417°E | 363 | *Ophrys candica* var. *minoa*, *Orchis anatolica* |
| 324 | Dumanlar | Antalya | 36.99785°N, 30.90477°E | 37 | *Himantoglossum robertianum* |
| 325 | Düzağaç | Antalya | 37.23460°N, 31.25631°E | 721 | – |
| 326 | Emişbeleni* | Antalya | 36.61346°N, 31.86730°E | 110 | *Epipactis helleborine* s.l., *Serapias bergonii* subsp. *politisii*, *Spiranthes spiralis* |
| 327 | Gazipaşa | Antalya | 36.28002°N, 32.30208°E | 31 | *Ophrys holoserica* subsp. *heterochila*, *Ophrys mammosa* subsp. *posteria* |
| 328 | Göynük Bld. | Antalya | 36.67218°N, 30.54400°E | 56 | – |
| 329 | Gözübüyük* | Antalya | 36.69999°N, 31.89088°E | 740 | *Anacamptis* sp., *Anacamptis* sp. 2, *Epipactis helleborine* s.l., *Limodorum abortivum*, *Ophrys* sp., *Spiranthes spiralis* |
| 330 | Hacıveliler | Antalya | 36.37742°N, 30.26817°E | 33 | – |
| 331 | Halitağalar | Antalya | 36.74648°N, 31.67780°E | 133 | *Serapias bergonii* subsp*. Politisii* |
| 332 | Halitağalar | Antalya | 36.74509°N, 31.66613°E | 113 | – |
| 333 | Halitağalar | Antalya | 36.73985°N, 31.64973°E | 147 | – |
| 334 | Hızırkahya | Antalya | 36.34671°N, 30.23629°E | 0 | – |
| 335 | Hocalar* | Antalya | 36.88937°N, 31.25082°E | 43 | *Anacamptis papilionacea* subsp*. messenica*, *Serapias bergonii* subsp*. politisii*, *Spiranthes spiralis* |
| 336 | İhsaniye | Antalya | 36.99415°N, 30.85506°E | 97 | *Anacamptis pyramidalis*, *Ophrys lutea* subsp*. minor*, *Ophrys mammosa* subsp*. posteria*, *Ophrys umbilicata* subsp*. umbilicata*, *Spiranthes spiralis* |
| 337 | İhsaniye | Antalya | 37.01008°N, 30.86299°E | 251 | *Spiranthes spiralis* |
| 338 | Ilıca | Antalya | 36.42664°N, 32.35410°E | 684 | *Ophrys holoserica* subsp*. heterochila*, *Orchis anatolica* |
| 339 | Ilıca | Antalya | 36.41092°N, 32.36840°E | 540 | *Limodorum abortivum*, *Ophrys mammosa* subsp. *Posteria* |
| 340 | Kadılar | Antalya | 36.71360°N, 31.63334°E | 36 | *Ophrys* sp., *Ophrys mammosa* subsp. *posteria*, *Ophrys tenthredinifera* subsp. *villosa*, *Orchis punctulata*, *Serapias bergonii* subsp. *politisii*, *Spiranthes spiralis* |
| 341 | Karabucak | Antalya | 37.10030°N, 31.26442°E | 495 | *Serapias bergonii* subsp. *Politisii* |
| 342 | Karabük* | Antalya | 37.14559°N, 31.19228°E | 133 | *Himantoglossum robertianum*, *Ophrys* sp., *Ophrys mammosa* subsp. *posteria*, *Ophrys tenthredinifera* subsp. *villosa*, *Orchis anatolica*, *Spiranthes spiralis* |
| 343 | Karabük | Antalya | 37.18254°N, 31.19455°E | 186 | *Himantoglossum robertianum*, *Ophrys holoserica* subsp. *heterochila*, *Ophrys lutea* subsp. *minor*, *Ophrys mammosa* subsp. *posteria*, *Spiranthes spiralis* |
| 344 | Karabük | Antalya | 37.19416°N, 31.20955°E | 379 | – |
| 345 | Karabük | Antalya | 37.20052°N, 31.21884°E | 441 | – |
| 346 | Karalar | Antalya | 36.36732°N, 32.34203°E | 324 | – |
| 347 | Karamanlar | Antalya | 36.71164°N, 31.82763°E | 571 | *Epipactis helleborine* s.l., *Limodorum abortivum* |
| 348 | Kargıcak | Antalya | 36.46099°N, 32.12150°E | 3 | *Ophrys mammosa* subsp. *posteria*, *Serapias bergonii* subsp. *politisii*, *Spiranthes spiralis* |
| 349 | Kaş | Antalya | 36.20386°N, 29.63725°E | 15 | – |
| 350 | Kasaba* | Antalya | 36.31133°N, 29.73116°E | 266 | *Anacamptis morio* subsp*. syriaca*, *Anacamptis papilionacea* subsp. *messenica*, *Limodorum abortivum*, *Ophrys* sp., *Ophrys lutea* subsp. *minor*, *Ophrys speculum* var. *orientalis*, *Ophrys subfusca* subsp. *cinereophila*, *Ophrys umbilicata* subsp. *umbilicata*, *Orchis anatolica*, *Serapias bergonii* subsp. *Politisii* |
| 351 | Kemer | Antalya | 36.61950°N, 30.55351°E | 7 | – |
| 352 | Kızılgüney | Antalya | 36.36019°N, 32.27863°E | 250 | *Limodorum abortivum* |
| 353 | Kızılgüney | Antalya | 36.37485°N, 32.31650°E | 189 | *Limodorum abortivum* |
| 354 | Kızılgüney | Antalya | 36.38487°N, 32.32288°E | 301 | *Limodorum abortivum* |
| 355 | Kumluca | Antalya | 36.35520°N, 30.29963°E | 24 | – |
| 356 | Kumluca | Antalya | 36.35606°N, 30.29984°E | 10 | – |
| 357 | Kumluca | Antalya | 36.38568°N, 30.27723°E | 44 | *Serapias bergonii* subsp. *Politisii* |
| 358 | Kumluca | Antalya | 36.37111°N, 30.27721°E | 5 | – |
| 359 | Macar | Antalya | 36.23203°N, 32.34481°E | 58 | *Ophrys holoserica* subsp. *heterochila*, *Spiranthes spiralis* |
| 360 | Mahmutlar | Antalya | 36.49721°N, 32.09322°E | 28 | – |
| 361 | Mavikent | Antalya | 36.32025°N, 30.33847°E | 26 | – |
| 362 | Okurcalar | Antalya | 36.65809°N, 31.70337°E | 68 | *Limodorum abortivum* |
| 363 | Orhanköy | Antalya | 36.70118°N, 31.86322°E | 120 | *Ophrys* sp., *Ophrys mammosa* subsp. *Posteria* |
| 364 | Örenşehir | Antalya | 36.70487°N, 31.66434°E | 110 | *Limodorum abortivum* |
| 365 | Pınarbaşı | Antalya | 36.24834°N, 29.68520°E | 691 | – |
| 366 | Saburlar | Antalya | 36.70914°N, 31.76789°E | 495 | *Anacamptis* sp., *Ophrys mammosa* subsp. *posteria*, *Serapias bergonii* subsp. *politisii*, *Spiranthes spiralis* |
| 367 | Sağırin* | Antalya | 36.99678°N, 31.20356°E | 55 | *Himantoglossum robertianum*, *Ophrys holoserica* subsp. *heterochila*, *Ophrys lutea* subsp. *minor*, *Ophrys mammosa* subsp. *posteria*, *Serapias bergonii* subsp. *politisii*, *Spiranthes spiralis* |
| 368 | Sağırin* | Antalya | 37.00989°N, 31.23050°E | 42 | *Anacamptis* sp., *Himantoglossum robertianum*, *Neotinea maculata*, *Ophrys lutea* subsp. *minor*, *Ophrys mammosa* subsp. *posteria*, *Ophrys speculum* var. *orientalis*, *Orchis italica*, *Serapias bergonii* subsp. *politisii*, *Spiranthes spiralis* |
| 369 | Sahilkılınçlı | Antalya | 36.19891°N, 29.77211°E | 293 | *Anacamptis morio* subsp. *syriaca*, *Ophrys lutea* subsp*. minor*, *Orchis anatolica*, *Serapias bergonii* subsp. *politisii*, *Spiranthes spiralis* |
| 370 | Sarıabalı* | Antalya | 36.98061°N, 31.20686°E | 36 | *Himantoglossum robertianum*, *Ophrys lutea* subsp. *minor*, *Ophrys mammosa* subsp. *posteria*, *Serapias bergonii* subsp. *politisii*, *Spiranthes spiralis* |
| 371 | Sarıcasu | Antalya | 36.40632°N, 30.28197°E | 93 | *Serapias bergonii* subsp. *Politisii* |
| 372 | Serik | Antalya | 36.91423°N, 31.08145°E | 110 | – |
| 373 | Side | Antalya | 36.77136°N, 31.42827°E | 25 | – |
| 374 | Side | Antalya | 36.78793°N, 31.40495°E | 119 | – |
| 375 | Solak | Antalya | 36.96246°N, 30.90088°E | 116 | – |
| 376 | Tasagil | Antalya | 36.92768°N, 31.22444°E | 54 | – |
| 377 | Taşağıl | Antalya | 36.91694°N, 31.23566°E | 37 | – |
| 378 | Tekirova | Antalya | 36.50430°N, 30.52307°E | 30 | – |
| 379 | Uğrak | Antalya | 36.36068°N, 32.23843°E | 115 | *Limodorum abortivum*, *Ophrys holoserica* subsp. *heterochila*, *Ophrys mammosa* subsp. *posteria*, *Spiranthes spiralis* |
| 380 | Uğrar* | Antalya | 36.28480°N, 29.70633°E | 216 | *Anacamptis morio* subsp. *syriaca*, *Limodorum abortivum*, *Ophrys lutea* subsp. *minor*, *Ophrys speculum* var. *orientalis*, *Ophrys subfusca* subsp. *cinereophila*, *Orchis anatolica*, *Orchis italica*, *Orchis simia*, *Serapias bergonii* subsp. *politisii* |
| 381 | Ulupınar* | Antalya | 36.42100°N, 30.42516°E | 383 | *Ophrys lutea* subsp. *minor*, *Ophrys umbilicata* subsp. *umbilicata*, *Serapias bergonii* subsp. *Politisii* |
| 382 | Yalçıdibi | Antalya | 36.70132°N, 31.67992°E | 109 | *Limodorum abortivum*, *Ophrys holoserica* subsp. *Episcopalis* |
| 383 | Yazır | Antalya | 36.42114°N, 30.42513°E | 383 | – |
| 384 | Yeşilköy | Antalya | 36.29473°N, 30.39841°E | 31 | *Himantoglossum robertianum*, *Serapias bergonii* subsp. *Politisii* |
| 385 | Yeşilköy | Antalya | 36.27184°N, 30.41221°E | 8 | *Ophrys candica* subsp. *lyciensis*, *Ophrys candica* var. *minoa*, *Spiranthes spiralis* |
| 386 | Yeşilyurt | Antalya | 36.97912°N, 31.08181°E | 35 | *Anacamptis papilionacea* subsp. *messenica*, *Anacamptis pyramidalis*, *Himantoglossum robertianum*, *Serapias bergonii* subsp. *politisii*, *Spiranthes spiralis* |
| 387 | Yukarıkocayatak* | Antalya | 36.93305°N, 30.96975°E | 19 | *Limodorum abortivum* |
| 388 | Bozdoğan | Aydın | 37.69763°N, 28.32558°E | 143 | *Anacamptis* sp., *Ophrys speculum* var. *orientalis*, *Orchis italica* |
| 389 | Direcik | Aydın | 37.83806°N, 28.26968°E | 54 | – |
| 390 | Yenipazar | Aydın | 37.82588°N, 28.21168°E | 48 | – |
| 391 | Ahmetbeyli | İzmir | 38.02897°N, 27.18689°E | 34 | – |
| 392 | Bademler | İzmir | 38.27627°N, 26.83159°E | 73 | *Anacamptis* sp., *Ophrys lutea* subsp. *minor*, *Ophrys speculum* var. *orientalis*, *Ophrys tenthredinifera* subsp. *villosa*, *Ophrys umbilicata* subsp. *Umbilicata* |
| 393 | Kavakdere | İzmir | 38.14196°N, 26.88890°E | 42 | *Himantoglossum robertianum* |
| 394 | Orta | İzmir | 38.04578°N, 27.05886°E | 0 | – |
| 395 | Ovacık | İzmir | 38.29910°N, 26.80521°E | 177 | – |
| 396 | Selçuk | İzmir | 37.94835°N, 27.35960°E | 1 | – |
| 397 | Urla | İzmir | 38.31825°N, 26.75525°E | 82 | – |
| 398 | Akcakavak | Muğla | 36.86079°N, 28.71465°E | 16 | *Ophrys holoserica* subsp. *heterochila*, *Spiranthes spiralis* |
| 399 | Balcılar | Muğla | 37.12506°N, 27.75755°E | 179 | *Anacamptis* sp., *Anacamptis morio* subsp*. caucasica*, *Anacamptis papilionacea* subsp. *messenica*, *Neotinea maculata*, *Ophrys holoserica* subsp. *heterochila*, *Ophrys reinholdii*, *Serapias bergonii* subsp. *politisii*, *Spiranthes spiralis* |
| 400 | Bayır | Muğla | 37.10906°N, 27.70012°E | 161 | *Anacamptis morio* subsp. *caucasica*, *Anacamptis papilionacea* subsp. *messenica*, *Himantoglossum robertianum*, *Ophrys bombyliflora*, *Ophrys holoserica* subsp. *heterochila*, *Ophrys lutea* subsp*. minor*, *Ophrys tenthredinifera* subsp. *villosa*, *Ophrys subfusca* subsp. *blithopertha*, *Neotinea maculata*, *Serapias bergonii* subsp. *politisii*, *Spiranthes spiralis* |
| 401 | Bayır* | Muğla | 36.71531°N, 28.19401°E | 371 | *Himantoglossum robertianum Ophrys holoserica* subsp. *heterochila*, *Ophrys lutea* subsp. *minor*, *Spiranthes spiralis* |
| 402 | Bayır | Muğla | 36.71288°N, 28.17974°E | 189 | – |
| 403 | Bayır | Muğla | 36.72886°N, 28.17599°E | 300 | *Neotinea maculate* |
| 404 | Beçin | Muğla | 37.25882°N, 27.80858°E | 161 | *Anacamptis papilionacea* subsp. *messenica*, *Anacamptis pyramidalis*, *Ophrys* sp., *Spiranthes spiralis* |
| 405 | Belceğiz | Muğla | 36.52524°N, 29.15746°E | 730 | *Ophrys holoserica* subsp. *heterochila*, *Orchis anatolica* |
| 406 | Bozalan | Muğla | 37.03541°N, 27.81938°E | 214 | *Himantoglossum robertianum*, *Ophrys holoserica* subsp. *heterochila*, *Ophrys lutea* subsp. *minor*, *Serapias bergonii* subsp. *Politisii* |
| 407 | Çakıralan 1/1* | Muğla | 37.12320°N, 27.91636°E | 144 | *Orchis anatolica*, *Ophrys lutea* subsp. *minor*, *Ophrys mammosa* subsp. *mammosa*, *Ophrys umbilicata* subsp. *umbilicata* |
| 408 | Çakıralan 1/2* | Muğla | 37.10484°N, 27.93409°E | 271 | *Anacamptis coriophora* subsp. *fragrans*, *Anacamptis papilionacea* subsp. *messenica*, *Ophrys sp.*, *Ophrys bombyliflora*, *Ophrys candica* var. *minoa*, *Ophrys holoserica* subsp. *heterochila*, *Ophrys lutea* subsp. *minor*, *Ophrys mammosa* subsp. *mammosa*, *Ophrys omegaifera* subsp. *omegaifera*, *Ophrys reinholdii*, *Ophrys speculum* var. *Orientalis*, *Ophrys tenthredinifera* subsp. *villosa*, *Serapias bergonii* subsp. *politisii*, *Spiranthes spiralis* |
| 409 | Çaltıözü* | Muğla | 36.55003°N, 29.34529°E | 54 | *Anacamptis* sp., *Anacamptis papilionacea* subsp. *messenica*, *Anacamptis pyramidalis*, *Himantoglossum robertianum*, *Serapias bergonii* subsp. *politisii*, *Spiranthes spiralis* |
| 410 | Çamlıbelen | Muğla | 37.25116°N, 27.90206°E | 488 | *Ophrys* sp., *Ophrys lutea* subsp. *minor*, *Ophrys mammosa* subsp. *mammosa*, *Orchis italica*, *Spiranthes spiralis* |
| 411 | Çamlık 1/1* | Muğla | 37.06205°N, 27.52845°E | 299 | *Himantoglossum robertianum*, *Ophrys holoserica* subsp. *heterochila*, *Spiranthes spiralis*, |
| 412 | Çamlık 1/2* | Muğla | 37.06965°N, 27.54928°E | 147 | *Anacamptis pyramidalis*, *Himantoglossum robertianum*, *Ophrys bombyliflora*, *Ophrys holoserica* subsp. *heterochila*, *Ophrys reinholdii*, *Ophrys umbilicata* subsp. *umbilicata*, *Orchis italica*, *Serapias bergonii* subsp. *bergonii* |
| 413 | Çamlık 1/3 | Muğla | 37.06854°N, 27.53828°E | 161 | *Ophrys holoserica* subsp. *heterochila*, *Ophrys reinholdii*, *Orchis italica*, *Serapias bergonii* subsp. *Politisii* |
| 414 | Çamurköy | Muğla | 36.53324°N, 29.35920°E | 68 | – |
| 415 | Çamurköy | Muğla | 36.54729°N, 29.38071°E | 110 | – |
| 416 | Dereköy* | Muğla | 37.09799°N, 27.94145°E | 178 | *Himantoglossum robertianum*, *Ophrys holoserica* subsp. *heterochila*, *Ophrys lutea* subsp. *minor*, *Ophrys mammosa* subsp. *mammosa*, *Serapias bergonii* subsp. *Politisii* |
| 417 | Döğüşbelen | Muğla | 36.97745°N, 28.59664°E | 112 | *Serapias bergonii* subsp. *Politisii* |
| 418 | Dörttepe* | Muğla | 37.15625°N, 27.64880°E | 73 | *Anacamptis pyramidalis*, *Himantoglossum robertianum*, *Ophrys bombyliflora*, *Ophrys holoserica* subsp. *heterochila*, *Ophrys lutea* subsp. *minor*, *Ophrys mammosa* subsp. *mammosa*, *Ophrys speculum* var. *orientalis*, *Ophrys tenthredinifera* subsp. *villosa*, *Ophrys umbilicata* subsp. *umbilicata*, *Orchis italica*, *Serapias bergonii* subsp. *politisii*, *Serapias orientalis* subsp. *carica*, *Spiranthes spiralis* |
| 419 | Dörttepe* | Muğla | 37.16560°N, 27.62280°E | 24 | *Anacamptis* sp., *Himantoglossum robertianum*, *Ophrys holoserica* subsp. *heterochila*, *Ophrys lutea* subsp. *minor*, *Ophrys mammosa* subsp*. mammosa*, *Ophrys tenthredinifera* subsp. *villosa*, *Ophrys umbilicata* subsp. *umbilicata*, *Serapias bergonii* subsp. *Politisii* |
| 420 | Eşen 1/1* | Muğla | 36.46842°N, 29.29045°E | 116 | *Anacamptis* sp., *Limodorum abortivum*, *Ophrys candica* var. *minoa*, *Ophrys holoserica* subsp. *heterochila*, *Ophrys lutea* subsp. *minor*, *Ophrys subfusca* subsp. *cinereophila*, *Orchis italica*, *Serapias bergonii* subsp. *politisii*, *Spiranthes spiralis* |
| 421 | Eşen 1/2* | Muğla | 36.45903°N, 29.28430°E | 116 | *Anacamptis* sp., *Anacamptis* sp. 2, *Ophrys* sp*.*, *Ophrys* sp. 2., *Serapias bergonii* subsp. *politisii* |
| 422 | Fethiye | Muğla | 36.61926°N, 29.12969°E | 13 | *Serapias bergonii* subsp. *politisii*, *Spiranthes spiralis* |
| 423 | Gökçeovacık | Muğla | 36.79678°N, 28.97513°E | 234 | *Himantoglossum robertianum*, *Limodorum abortivum*, *Ophrys holoserica* subsp. *heterochila*, *Ophrys lutea* subsp. *minor*, *Ophrys reinholdii*, *Orchis italica*, *Serapias bergonii* subsp. *bergonii*, *Spiranthes spiralis* |
| 424 | Gölbent* | Muğla | 36.41699°N, 29.26921°E | 104 | *Anacamptis* sp., *Ophrys* sp., *Serapias bergonii* subsp. *politisii*, *Spiranthes spiralis* |
| 425 | Gürköy | Muğla | 36.79339°N, 28.82735°E | 51 | *Himantoglossum robertianum*, *Ophrys holoserica* subsp. *heterochila*, *Serapias bergonii* subsp. *politisii*, *Spiranthes spiralis* |
| 426 | Hacıosmanlar* | Muğla | 36.51356°N, 29.39147°E | 160 | *Anacamptis* sp., *Anacamptis coriophora* subsp. *fragrans*, *Anacamptis papilionacea* subsp. *messenica*, *Anacamptis pyramidalis*, *Himantoglossum robertianum*, *Neotinea maculata*, *Ophrys bombyliflora*, *Ophrys lutea* subsp. *minor*, *Ophrys subfusca* subsp*. cinereophila*, *Ophrys speculum* var. *orientalis*, *Serapias bergonii* subsp. *politisii*, *Spiranthes spiralis* |
| 427 | Hasanlar | Muğla | 37.12632°N, 27.76482°E | 173 | *Himantoglossum robertianum*, *Ophrys holoserica* subsp. *heterochila*, *Ophrys lutea* subsp. *minor* |
| 428 | Hisarönü | Muğla | 36.82362°N, 28.13778°E | 61 | *Anacamptis pyramidalis*, *Ophrys holoserica* subsp. *episcopalis*, *Spiranthes spiralis* |
| 429 | Hisarönü* | Muğla | 36.79720°N, 28.14670°E | 58 | *Anacamptis pyramidalis*, *Ophrys holoserica* subsp. *episcopalis*, *Ophrys holoserica* subsp. *heterochila*, *Ophrys mammosa* subsp. *mammosa*, *Spiranthes spiralis* |
| 430 | İkizköy* | Muğla | 37.16931°N, 27.85229°E | 173 | *Anacamptis* sp., *Cephalanthera epipactoides*, *Ophrys* sp., *Ophrys holoserica* subsp. *heterochila*, *Ophrys lutea* subsp. *minor*, *Ophrys mammosa* subsp. *mammosa*, *Ophrys speculum* var. *orientalis*, *Spiranthes spiralis* |
| 431 | İslamlar | Muğla | 39.11674°N, 26.89386°E | 20 | – |
| 432 | Kalınağıl | Muğla | 37.25328°N, 27.87901°E | 422 | *Anacamptis* sp*.*, *Ophrys mammosa* subsp*. Mammosa* |
| 433 | Kalınağıl | Muğla | 37.24411°N, 27.86157°E | 381 | *Anacamptis morio* subsp*. caucasica*, *Neotinea maculata*, *Ophrys* sp., *Ophrys speculum* var. *orientalis*, *Spiranthes spiralis* |
| 434 | Karacahisar | Muğla | 37.14985°N, 27.80752°E | 300 | *Anacamptis* sp., |
| 435 | Karacaören* | Muğla | 36.82434°N, 28.99022°E | 665 | *Anacamptis morio* subsp. *caucasica*, *Cephalanthera epipactoides*, *Himantoglossum robertianum*, *Limodorum abortivum*, *Neotinea maculata*, *Ophrys* sp., *Ophrys holoserica* subsp. *heterochila*, *Ophrys reinholdii*, *Orchis anatolica*, *Spiranthes spiralis* |
| 436 | Kayadibi | Muğla | 36.49256°N, 29.40256°E | 191 | *Ophrys holoserica* subsp*. episcopalis*, *Ophrys iricolor*, *Ophrys subfusca* subsp. *cinereophila*, *Ophrys umbilicata* subsp. *umbilicata*, *Serapias bergonii* subsp. *Politisii* |
| 437 | Kınık | Muğla | 36.35326°N, 29.32393°E | 16 | – |
| 438 | Kısırlar* | Muğla | 37.12140°N, 27.74763°E | 182 | *Himantoglossum robertianum*, *Ophrys holoserica* subsp. *heterochila*, *Ophrys mammosa* subsp. *mammosa*, *Ophrys subfusca* subsp. *blithopertha*, *Ophrys tenthredinifera* subsp. *villosa*, *Serapias bergonii* subsp. *bergonii*, *Serapias bergonii* subsp. *politisii*, *Orchis italica*, *Spiranthes spiralis* |
| 439 | Kızılağaç 1/1* | Muğla | 37.03038°N, 27.49962°E | 75 | *Anacamptis coriophora* subsp. *fragrans*, *Anacamptis papilionacea* subsp. *messenica*, *Anacamptis pyramidalis*, *Himantoglossum robertianum*, *Limodorum abortivum*, *Ophrys argolica* subsp. *lucis*, *Ophrys ferrum-equinum* subsp. *ferrum-equinum*, *Ophrys ferrum-equinum* subsp. *labiosa*, *Ophrys holoserica* subsp. *heterochila*, *Ophrys holoserica* subsp. *homeri*, *Ophrys iricolor*, *Ophrys lutea* subsp. *minor*, *Ophrys speculum* var. *orientalis*, *Ophrys tenthredinifera* subsp. *villosa*, *Orchis italica*, *Serapias orientalis* subsp. *carica*, *Serapias bergonii* subsp. *bergonii*, *Serapias bergonii* subsp. *politisii*, *Spiranthes spiralis* |
| 440 | Kızılağaç 1/2* | Muğla | 37.04483°N, 27.47854°E | 100 | *Anacamptis morio* subsp. *caucasica*, *Ophrys ferrum-equinum* subsp. *ferrum-equinum*, *Ophrys holoserica* subsp. *heterochila*, *Ophrys subfusca* subsp. *cinereophila*, *Ophrys umbilicata* subsp. *umbilicata*, *Orchis italica*, *Serapias bergonii* subsp. *bergonii*, *Serapias bergonii* subsp. *politisii*, *Serapias orientalis* subsp. *carica* |
| 441 | Kızılağaç 2/1* | Muğla | 37.34058°N, 27.55581°E | 83 | *Anacamptis* sp., *Himantoglossum robertianum*, *Ophrys lutea* subsp. *minor*, *Ophrys mammosa* subsp. *mammosa*, *Ophrys speculum* var. *orientalis*, *Ophrys umbilicata* subsp. *umbilicata*, *Orchis italica*, *Serapias bergonii* subsp. *politisii*, *Spiranthes spiralis* |
| 442 | Ortaca | Muğla | 36.83469°N, 28.77213°E | 17 | – |
| 443 | Ortakentyahşi | Muğla | 37.05227°N, 27.34809°E | 21 | – |
| 444 | Osmaniye | Muğla | 36.76022°N, 28.21113°E | 524 | *Orchis provincialis* |
| 445 | Ovakışlacık | Muğla | 37.30338°N, 27.63178°E | 267 | *Himantoglossum robertianum* |
| 446 | Ölüdeniz | Muğla | 36.57873°N, 29.15380°E | 321 | *Anacamptis pyramidalis*, *Ophrys holoserica* subsp. *episcopalis*, *Orchis anatolica*, *Serapias bergonii* subsp. *politisii*, *Spiranthes spiralis* |
| 447 | Sarıgerme* | Muğla | 36.71888°N, 28.71070°E | 7 | *Himantoglossum robertianum*, *Ophrys holoserica* subsp. *heterochila*, *Orchis italica*, *Serapias bergonii* subsp. *politisii*, *Spiranthes spiralis* |
| 448 | Sazköy* | Muğla | 37.09499°N, 27.62934°E | 60 | *Anacamptis papilionacea* subsp. *messenica*, *Anacamptis sancta*, *Himantoglossum robertianum*, *Ophrys bombyliflora*, *Ophrys lutea* subsp. *minor*, *Ophrys speculum* var. *Orientalis*, *Ophrys tenthredinifera* subsp. *villosa*, *Ophrys umbilicata* subsp. *umbilicata*, *Spiranthes spiralis* |
| 449 | Sazköy | Muğla | 37.08905°N, 27.61345°E | 24 | *Anacamptis* sp., *Himantoglossum robertianum*, *Ophrys lutea* subsp. *minor*, *Ophrys tenthredinifera* subsp. *villosa*, *Orchis italica*, *Serapias bergonii* subsp. *politisii*, *Spiranthes spiralis* |
| 450 | Söğütcük | Muğla | 37.13187°N, 27.77432°E | 220 | *Anacamptis* sp., *Himantoglossum robertianum* |
| 451 | Söke | Muğla | 37.73160°N, 27.39650°E | 48 | – |
| 452 | Söke | Muğla | 37.77133°N, 27.42992°E | 53 | – |
| 453 | Tepearası | Muğla | 36.87082°N, 28.69699°E | 10 | *Ophrys holoserica* subsp. *heterochila*, *Spiranthes spiralis* |
| 454 | Tepearası | Muğla | 36.86413°N, 28.69641°E | 46 | *Anacamptis* sp., *Ophrys* sp., *Ophrys holoserica* subsp. *heterochila*, *Spiranthes spiralis* |
| 455 | Yanıklar | Muğla | 36.69974°N, 29.06088°E | 38 | *Ophrys holoserica* subsp. *heterochila*, *Ophrys holoserica* subsp. *episcopalis*, *Spiranthes spiralis* |

**Table S2**

Plant characteristics of studied taxa based on herbarium specimens and *literature data.

| **Name of species** | **Measured herbarium specimens** | **No. measured herbarium specimens** | **Mean height of flowering stems**  **± SD (mm)** | **Mean lenght of inflorescence ± SD (mm)** | **Mean number of flowers ± SD (mm)** | **Mean flowering season (based on Kreutz and Çolak, 2009)*** | **Mean new**  **tuber width**  **± SD (mm)** | **Mean new**  **tuber lenght**  **± SD (mm)** | **Mean new tuber volume ± SD (cm^3^)** |
| --- | --- | --- | --- | --- | --- | --- | --- | --- | --- |
| *Anacamptis coriophora* subsp. *fragrans* | *Anacamptis coriophora* subsp. *fragrans* | 8 | 223.3±100.6 | 58.3±37.5 | 22.4±10.7 | 14.0 | 12.3 ± 4.3 | 16.8 ± 6.5 | 1.8 ± 2.1 |
| *Anacamptis morio* subsp. *caucasica* | *Anacamptis morio* agg. | 25 | 139.9 ± 43.3 | 42.6 ± 9.3 | 8.5 ± 2.2 | 11.5 | 10.4 ± 2.1 | 14.2 ± 2.5 | 0.9 ± 0.4 |
| *Anacamptis morio* subsp. *syriaca* | *Anacamptis morio* subsp. *syriaca* | 4 | 103.3 ± 14.5 | 29.0 ± 6.7 | 4.3 ± 1.0 | 12.5 | 9.5 ± 2.1 | 12.3 ± 2.1 | 0.6 ± 0.3 |
| *Anacamptis papilionacea* subsp. *messenica* | *Anacamptis papilionacea* agg. | 33 | 140.8 ± 50.1 | 38.4 ± 9.5 | 4.3 ± 1.9 | 9.0 | 9.4 ± 3.1 | 14.6 ± 3.6 | 0.8 ± 0.7 |
| *Anacamptis pyramidalis* | *Anacamptis pyramidalis* | 28 | 286.3 ± 83.5 | 39.9 ± 15.0 | 23.5 ± 10.3 | 15.0 | 9.6 ± 2.6 | 15.5 ± 4.3 | 0.9 ± 0.6 |
| *Anacamptis sancta* | *Anacamptis sancta* | 15 | 239.7 ± 94.2 | 70.9 ± 27.2 | 17.9 ± 9.1 | 14.5 | 11.9 ± 2.9 | 17.1 ± 5.9 | 1.5 ± 1.6 |
| *Anacamptis* sp. | *Anacamptis* sp. | 232 | 213.4 ± 103.5 | 56.2 ± 34.7 | 12.8 ± 9.3 | 12.6 | 10.6 ± 3.0 | 15.4 ± 4.6 | 1.1 ± 1.0 |
| *Himantoglossum caprinum* | *Himantoglossum caprinum* | 7 | 555.7 ± 123.1 | 228.3 ± 65.9 | 19.4 ± 8.0 | 17.0 | 24.1 ± 4.5 | 42.0 ± 9.7 | 13.8 ± 8.0 |
| *Himantoglossum comperianum* | *Himantoglossum comperianum* | 9 | 390.9 ± 64.6 | 126.3 ± 38.3 | 9.7 ± 4.1 | 17.0 | 16.9 ± 6.1 | 26.7 ± 9.7 | 5.4 ± 6.8 |
| *Himantoglossum jankae* | *Himantoglossum jankae* | 4 | 476.5 ± 116.0 | 140.5 ± 89.1 | 14.5 ± 4.2 | 19.0 | 13.3 ± 2.5 | 18.0 ± 6.4 | 1.8 ± 0.9 |
| *Himantoglossum montis-taurii* | *Himantoglossum* sp. | 11 | 526.9 ± 121.3 | 196.4 ± 83.3 | 17.6 ± 7.1 | 16.0 | 20.2 ± 6.6 | 33.3 ± 14.0 | 9.4 ± 8.7 |
| *Himantoglossum robertianum* | *Himantoglossum robertianum* | 13 | 324.9 ± 112.1 | 117.6 ± 44.5 | 27.7 ± 16.6 | 8.0 | 20.3 ± 9.2 | 30.2 ± 9.1 | 9.1 ± 9.7 |
| *Neotinea maculata* | *Neotinea maculata* | 20 | 170.1 ± 37.9 | 37.7 ± 13.4 | 14.9 ± 5.1 | 12.0 | 9.0 ± 2.8 | 15.1 ± 4.4 | 0.8 ± 0.7 |
| *Ophrys amanensis* subsp. *antalyensis* | *Ophrys* sp. | 218 | 169.5 ± 79.4 | 56.2 ± 35.3 | 3.5 ± 1.8 | 15.0 | 10.8 ± 3.4 | 14.4 ± 4.7 | 1.1 ± 1.1 |
| *Ophrys apifera* | *Ophrys apifera* | 16 | 238.7 ± 82.8 | 66.3 ± 23.0 | 3.4 ± 1.2 | 13.0 | 11.1 ± 3.3 | 14.6 ± 3.2 | 1.1 ± 0.9 |
| *Ophrys argolica* subsp. *lucis* | *Ophrys argolica* subsp. *lucis* | 8 | 175.1 ± 56.6 | 52.0 ± 25.7 | 3.0 ± 1.1 | 9.0 | 10.1 ± 3.3 | 14.1 ± 4.8 | 0.9 ± 0.8 |
| *Ophrys bombyliflora* | *Ophrys bombyliflora* | 10 | 98.2 ± 26.9 | 36.4 ± 16.1 | 2.6 ± 0.8 | 11.0 | 9.6 ± 2.8 | 11.9 ± 3.7 | 0.7 ± 0.6 |
| *Ophrys candica* var. *Minoa* | *Ophrys* sp. | 218 | 169.5 ± 79.4 | 56.2 ± 35.3 | 3.5 ± 1.8 | 12.0 | 10.8 ± 3.4 | 14.4 ± 4.7 | 1.1 ± 1.1 |
| *Ophrys heldreichii* subsp. *calypsus* | *Ophrys* sp. | 218 | 169.5 ± 79.4 | 56.2 ± 35.3 | 3.5 ± 1.8 | 11.0 | 10.8 ± 3.4 | 14.4 ± 4.7 | 1.1 ± 1.1 |
| *Ophrys holoserica* subsp. *episcopalis* | *Ophrys* sp. | 218 | 169.5 ± 79.4 | 56.2 ± 35.3 | 3.5 ± 1.8 | 14.0 | 10.8 ± 3.4 | 14.4 ± 4.7 | 1.1 ± 1.1 |
| *Ophrys holoserica* subsp. *heterochila* | *Ophrys* sp. | 218 | 169.5 ± 79.4 | 56.2 ± 35.3 | 3.5 ± 1.8 | 8.5 | 10.8 ± 3.4 | 14.4 ± 4.7 | 1.1 ± 1.1 |
| *Ophrys holoserica* subsp. *homeri* | *Ophry* sp. | 218 | 169.5 ± 79.4 | 56.2 ± 35.3 | 3.5 ± 1.8 | 12.0 | 10.8 ± 3.4 | 14.4 ± 4.7 | 1.1 ± 1.1 |
| *Ophrys lutea* subsp. *minor* | *Ophrys lutea* agg. | 30 | 137.9 ± 51.7 | 41.1 ± 21.7 | 3.4 ± 1.6 | 10.0 | 9.3 ± 3.4 | 12.5 ± 4.0 | 0.8 ± 1.0 |
| *Ophrys mammosa subsp. mammosa* | *Ophrys mammosa* agg. | 28 | 164.0 ± 53.1 | 47.6 ± 21.7 | 3.3 ± 2.4 | 9.5 | 10.0 ± 3.1 | 12.1 ± 4.8 | 0.9 ± 1.2 |
| *Ophrys oestrifera* subsp. *oestrifera* | *Ophrys oestrifera* agg. | 8 | 189.1 ± 30.8 | 55.8 ± 17.1 | 4.8 ± 1.0 | 13.5 | 12.3 ± 3.3 | 18.0 ± 3.7 | 1.6 ± 0.9 |
| *Ophrys phrygia* | *Ophrys phrygia* | 7 | 197.0 ± 69.7 | 75.3 ± 50.1 | 4.3 ± 2.4 | 15.5 | 10.6 ± 2.0 | 14.4 ± 1.6 | 0.9 ± 0.4 |
| *Ophrys* sp. | *Ophrys* sp. | 218 | 169.5 ± 79.4 | 56.2 ± 35.3 | 3.5 ± 1.8 | 15.0 | 10.8 ± 3.4 | 14.4 ± 4.7 | 1.1 ± 1.1 |
| *Ophrys speculum* var. *Orientalis* | *Ophrys speculum* agg. | 17 | 145.2 ± 50.5 | 48.5 ± 19.5 | 3.9 ± 1.6 | 10.0 | 12.1 ± 3.5 | 17.8 ± 6.2 | 1.7 ± 1.4 |
| *Ophrys strausii* subsp. *strausii* | *Ophrys* sp. | 218 | 169.5 ± 79.4 | 56.2 ± 35.3 | 3.5 ± 1.8 | 13.5 | 10.8 ± 3.4 | 14.4 ± 4.7 | 1.1 ± 1.1 |
| *Ophrys subfusca* subsp. *blitopertha* | *Ophrys* sp. | 218 | 169.5 ± 79.4 | 56.2 ± 35.3 | 3.5 ± 1.8 | 10.5 | 10.8 ± 3.4 | 14.4 ± 4.7 | 1.1 ± 1.1 |
| *Ophrys tenthredinifera* subsp. *villosa* | *Ophrys tenthredinifera* agg. | 19 | 124.8 ± 51.8 | 42.7 ± 19.9 | 2.7 ± 1.3 | 9.0 | 12.5 ± 3.2 | 15.2 ± 4.3 | 1.5 ± 1.3 |
| *Ophrys ulupinara* | *Ophrys* sp. | 218 | 169.5 ± 79.4 | 56.2 ± 35.3 | 3.5 ± 1.8 | 10.0 | 10.8 ± 3.4 | 14.4 ± 4.7 | 1.1 ± 1.1 |
| *Ophrys umbilicata* subsp. *umbilicata* | *Ophrys umbilicata* agg. | 12 | 128.4 ± 32.3 | 51.5 ± 15.6 | 3.8 ± 1.6 | 10.5 | 10.0 ± 2.9 | 15.3 ± 5.0 | 0.9 ± 0.8 |
| *Orchis anatolica* | *Orchis anatolica* | 110 | 182.8 ± 56.5 | 58.8 ± 23.4 | 6.1 ± 3.7 | 12.0 | 8.9 ± 2.8 | 14.4 ± 4.4 | 0.7 ± 0.7 |
| *Orchis italica* | *Orchis italica* | 22 | 216.7 ± 43.3 | 51.4 ± 12.4 | 16.5 ± 5.3 | 10.5 | 12.0 ± 3.3 | 20.3 ± 4.3 | 1.7 ± 1.1 |
| *Orchis simia* | *Orchis simia* | 12 | 232.0 ± 39.5 | 43.1 ± 10.7 | 20.7 ± 6.3 | 13.0 | 12.4 ± 3.1 | 19.3 ± 3.7 | 1.7 ± 1.1 |
| *Platanthera chlorantha* subsp. *chlorantha* | *Platanthera chlorantha* agg. | 13 | 431.3 ± 109.0 | 138.8 ± 48.3 | 22.2 ± 9.6 | 18.0 | 11.1 ± 2.9 | 26.6 ± 7.1 | 1.8 ± 1.2 |
| *Serapias bergonii* subsp. *politisii* | *Serapias* sp. | 41 | 213.9 ± 83.7 | 76.0 ± 34.9 | 3.9 ± 1.7 | 11.8 | 11.2 ± 2.9 | 17.0 ± 5.0 | 1.3 ± 1.1 |
| *Spiranthes spiralis* | *Spiranthes spiralis* | 26 | 169.6 ± 56.6 | 62.0 ± 24.5 | 16.5 ± 5.3 | 27.5 | 10.0 ± 2.6 | 28.1 ± 10.9 | 1.7 ± 1.7 |
| *Steveniella satyrioides* | *Steveniella satyrioides* | 5 | 211.6 ± 27.4 | 67.0 ± 20.2 | 8.8 ± 3.0 | 13.0 | 8.8 ± 1.3 | 16.8 ± 6.8 | 0.8 ± 0.6 |
| *Anacamptis collina* | *Anacamptis* sp. | 8 | 183.1 ± 47.7 | 74.5 ± 29.2 | 10.4 ± 4.1 | 8.5 | 9.8 ± 3.1 | 15.8 ± 3.2 | 0.9 ± 0.9 |
| *Anacamptis coriophora* agg. | *Anacamptis* sp. | 19 | 213.7 ± 47.2 | 50.4 ± 13.3 | 20.8 ± 5.5 | 16.0 | 11.1 ± 2.7 | 15.5 ± 5.1 | 1.1 ± 0.8 |
| *Anacamptis laxiflora* agg. | *Anacamptis* sp. | 43 | 323.8 ± 119.7 | 104.7 ± 43.1 | 14.8 ± 9.1 | 13.5 | 11.8 ± 2.8 | 17.6 ± 4.7 | 1.4 ± 1.0 |
| *Anacamptis morio* subsp. *picta* | *Anacamptis* sp. | 49 | 165.6 ± 55.4 | 38.7 ± 15.7 | 7.9 ± 3.3 | 11.0 | 10.2 ± 3.2 | 13.8 ± 4.5 | 1.0 ± 1.0 |
| *Ophrys ferrum-equinum* agg. | *Ophrys* sp. | 8 | 274.4 ± 133.4 | 114.6 ± 63.0 | 3.9 ± 2.0 | 10.0 | 11.0 ± 3.2 | 15.8 ± 4.3 | 1.2 ± 1.1 |
| *Ophrys reinholdii* | *Ophrys* sp. | 6 | 282.8 ± 188.6 | 108.7 ± 97.7 | 5.8 ± 3.1 | 10.5 | 14.5 ± 5.4 | 19.7 ± 6.3 | 2.7 ± 2.0 |
| *Ophrys sphegodes* | *Ophrys* sp. | 28 | 209.8 ± 67.1 | 73.0 ± 1.5 | 3.6 ± 35.5 | 9.5 | 12.2 ± 3.1 | 15.8 ± 3.9 | 1.4 ± 0.9 |
| *Ophrys fusca* agg. | *Ophrys* sp. | 16 | 135.8 ± 56.8 | 46.3 ± 22.8 | 3.4 ± 1.7 | 10.8 | 8.9 ± 3.4 | 11.4 ± 3.1 | 0.6 ± 0.7 |
| *Ophrys iricolor* | *Ophrys* sp. | 5 | 160.4 ± 53.5 | 54.6 ± 29.0 | 2.4 ± 1.5 | 10.0 | 11.6 ± 3.2 | 12.6 ± 2.3 | 1.0 ± 0.5 |
| *Ophrys argolica* subsp. *lucis* | *Ophrys* sp. | 8 | 175.1 ± 56.6 | 52.0 ± 25.7 | 3.0 ± 1.1 | 9.0 | 10.1 ± 3.3 | 14.1 ± 4.8 | 0.9 ± 0.8 |
| *Serapias laxiflora* | *Serapias* sp. | 19 | 230.2 ± 99.5 | 83.5 ± 43.0 | 3.8 ± 1.9 | 11.0 | 11.4 ± 3.2 | 16.3 ± 5.3 | 1.3 ± 1.3 |
| *Serapias orientalis* | *Serapias* sp. | 8 | 147.0 ± 22.3 | 53.1 ± 14.8 | 3.8 ± 1.8 | 10.5 | 11.4 ± 2.4 | 16.4 ± 3.7 | 1.2 ± 0.8 |
| *Serapias parviflora* | *Serapias* sp. | 5 | 251.6 ± 61.0 | 84.0 ± 31.1 | 3.4 ± 0.9 | 12.5 | 9.8 ± 2.7 | 16.6 ± 4.1 | 1.0 ± 0.8 |
| *Serapias vomeracea* | *Serapias* sp. | 9 | 7.9 ± 66.8 | 17.8 ± 23.8 | 0.7 ± 1.3 | 13.0 | 218.1 ± 2.9 | 76.1 ± 5.7 | 4.3 ± 1.2 |
